# Integrated network pharmacology and clinical study to reveal the efficacy and tolerability of Huoxue Tongjiang decoction for the treatment of non-erosive reflux disease-related symptoms

**DOI:** 10.1515/jtim-2025-0043

**Published:** 2025-10-16

**Authors:** Yanping Tang, Peicai Li, Yanxia Gong, Lei Liu, Jiannan Jia, Lei Yang

**Affiliations:** Department of Gastroenterology, Tianjin Integrated Traditional Chinese and Western Medicine Hospital, Tianjin Nankai Hospital; Tianjin University, Tianjin, China; Tianjin Key Laboratory of Acute Abdomen Disease Associated Organ Injury and ITCWM Repair, Tianjin Integrated Traditional Chinese and Western Medicine Hospital, Tianjin Nankai Hospital; Tianjin University, Tianjin, China

**Keywords:** non-erosive reflux disease, huoxue tongjiang decoction, randomized controlled trial, double-Blind, placebo control

## Abstract

**Background and objectives:**

Non-erosive reflux disease (NERD), a common gastrointestinal disorder, often responds poorly to acid suppressants. This study evaluated the efficacy and safety of Huoxue Tongjiang Decoction (HTD), a patented traditional Chinese medicine (TCM), for NERD with Qi stagnation-blood stasis syndrome, integrating network pharmacology and clinical trials.

**Methods:**

Network pharmacology identified HTD's active components and targets using TCMSP and GeneCards databases. A "drug-component-target" network was constructed, followed by protein interaction analysis, Gene body analysis (GO), and KEGG pathway enrichment via DAVID. A randomized, double-blind, placebo-controlled trial enrolled 64 NERD patients with Qi stagnation-blood stasis syndrome, randomized to HTD (*n* = 32) or placebo (*n* = 32) for 4 weeks, with 2-week follow-up. Outcomes included reflux/heartburn severity (Visual Analog Scale [VAS], gastroesophageal reflux disease questionnaire [GERD-Q]), TCM syndrome scores, SF-36 quality-of-life, HAD scores, and recurrence rates.

**Results:**

Network analysis revealed 20 core HTD components (*e.g*., quercetin, luteolin, baicalin) and 11 key targets (TNF, IL6, TP53), primarily modulating HIF-1 and TNF signaling pathways. Clinically, 30 HTD and 31 placebo patients completed the study. After 2/4 weeks and follow-up, HTD showed significantly higher overall symptom remission (43.3%, 73.3%, 73.3%) *versus* placebo (16.1% at all timepoints; *P* < 0.05). Reflux response rates for HTD were 30.0%, 66.7%, 66.7% *vs*. 12.9% (placebo; *P* < 0.0001). Heartburn response rates in HTD were 43.3%, 63.3%, 76.7% *vs*. 16.1% (*P* < 0.05). VAS reflux scores decreased from 3.75 ± 1.599 to 1.98 ± 1.292 (HTD) *vs*. minimal changes in placebo; heartburn scores improved similarly (3.33 ± 1.724 to 1.81 ± 1.471; *P* < 0.05 post-2 weeks). GERD-Q scores were lower in HTD (7.9 ± 2.29, 6.2 ± 2.07, 6.8 ± 2.40) . placebo (9.4 ± 2.83, 8.9 ± 2.55, 8.9 ± 2.59; *P* < 0.05). TCM syndrome improvement rates in HTD were 56.7%, 86.7%, 76.7% *vs*. 16.1%, 25.8%, 41.9% (*P* < 0.05). SF-36 physical pain, energy, and mental health scores improved in HTD (*P* < 0.05). Recurrence rates were 27.3% (HTD) *vs*. 40.0% (placebo; *P* < 0.05). No adverse events occurred.

**Conclusions:**

HTD significantly alleviates reflux, heartburn, and retrosternal pain in NERD, improves quality of life, reduces recurrence, and demonstrates duration-dependent efficacy. Its anti-inflammatory mechanisms may involve TNF and HIF-1 pathways, with no safety concerns observed.

## Introduction

Gastroesophageal reflux disease (GERD) is characterized by symptoms and complications resulting from the reflux of gastric contents into the esophagus.^[1]^ GERD includes three clinical subtypes: non-erosive reflux disease (NERD), erosive esophagitis (RE), and Barrett’s esophagus (BE).^[2]^ NERD is defined by typical gastroesophageal reflux symptoms, such as reflux and heartburn, in the absence of esophageal mucosal erosion detected through endoscopy. GERD is a prevalent digestive disorder, affecting 8%–33% of the global population, with its incidence rising by 77.53% between 1990 and 2019.^[3]^ NERD accounts for approximately 70% of GERD cases.^[4, 5]^

Current GERD management is challenging. Oral antacids, including proton pump inhibitors (PPIs) and potassium-competitive acid blockers, are the first-line treatment. However, 10%–40% of patients fail to respond to antacid therapy.^[6]^ The efficacy of antacids is notably lower in NERD, with about 50% of patients experiencing persistent symptoms despite standard-dose PPI treatment,^[7]^ and a recurrence rate exceeding 50% upon drug withdrawal.^[8]^ Furthermore, the optimal PPI treatment duration remains unclear, necessitating long-term maintenance therapy for many patients. Prolonged antacid use is associated with potential adverse effects, including increased risks of fractures, malnutrition, dementia, and hypergastrinemiarelated complications.^[9]^ Frequent GERD relapses not only impair patients’ psychological well-being and quality of life but also impose significant healthcare costs due to recurrent consultations.^[10, 11]^ Thus, exploring alternative therapies for effective NERD management is clinically imperative. The pathogenesis of NERD is multifactorial, involving pathological acid exposure, esophageal mucosal barrier dysfunction, visceral hypersensitivity, and other factors.^[12, 13, 14]^ Additionally, 24-hour esophageal pH-impedance monitoring has demonstrated that weakly acidic reflux can induce both typical and atypical GERD symptoms.^[15]^ Weak acid reflux, non-acid reflux, gastrointestinal motility disorders, and visceral hypersensitivity-related NERD symptoms often respond poorly to PPIs, with no effective treatment currently available in Western medicine.

TCM offers a promising therapeutic alternative by employing individualized and holistic treatment approaches. According to TCM theory, the fundamental pathogenesis of NERD involves “stomach imbalance and reversed stomach Qi”. Qi stagnation, damp heat, and blood stasis are key pathological components of NERD. Among these, Qi stagnation and blood stasis syndrome are commonly observed in NERD.

Qi stagnation is a syndrome manifested by the obstruction of Qi movement in the zang-fu organs and meridians, resulting in poor circulation. Damp heat is a syndrome caused by the interweaving of damp and heat pathogens, leading to digestive dysfunction. Blood stasis is a syndrome characterized by internal obstruction of blood. Qi stagnation often leads to poor blood circulation and the formation of blood stasis.^[16]^ Based on extensive clinical practice, our research team developed the Huoxue Tongjiang Decoction (HTD), which promotes blood circulation, regenerates tissue, relieves stomach reflux, soothes the liver, and regulates Qi. This formula demonstrated significant clinical efficacy in treating NERD with Qi stagnation and blood stasis syndrome, earning a Chinese invention patent in 2023. Preclinical studies have shown that HTD reduces gastric acid secretion, increases lower esophageal sphincter pressure, enhances gastric emptying, and alleviates esophageal visceral hypersensitivity.^[17, 18, 19, 20]^ To further validate its clinical utility, we conducted a double-blind, placebo-controlled study to evaluate the efficacy, quality-of-life impact, and safety of HTD in treating NERD with Qi stagnation and blood stasis syndrome.

## Materials and methods

### Activity screening and target collection of HTD

By searching the pharmacology database and analysis platform of TCM System (TCMSP, http://lsp.nwu.edu.cn/tcmsp.php) *via* screening blood drop party formula in white and, pinellia, salvia miltiorrhiza, inula flower, sweet, bitter orange, and turmeric Oral bioavailability (OB) ≥30% and drug-like index (DL) ≥0.18 were used as screening conditions to obtain the active ingredients. Then click the “active ingredient” option in TCMSP to obtain the protein target information corresponding to the active ingredient; After the obtained drug target information was combined with weight loss, the Unipot database (https://www.uniprot.org/) was used to standardize the protein names, and the species were screened as homo sapiens, validated “reviewed” targets, The standard official gene symbol is the potential target of HTD.

### Screening of common targets of HTD and NERD

Retrieval of NERD in GeneCards database (https://www.Genecards.org/); Venny2.1 software was used to collect the intersection of the drug targets of HTD and the disease targets of NERD as the potential targets of HTD for the treatment of NERD.

### Protein interaction network construction of HTD for the treatment of NERD

Upload the common targets of HTD and NERD to STRING database (https://string-db.org/), select species “Homo sapiens” and set the lowest protein interaction threshold “Highest confidence≥0.9”. Other parameters were set unchanged, single node in the network was removed, and protein interaction information was obtained. The obtained interaction parameters are imported into Cytoscape3.8.2 software to construct a PPI Network diagram, and the Network structure is topological analyzed by the “Network Analyzer” plug-in. According to betweenness centrality, to get the core target by screening of Total Centrality (BC), closeness (CC) and node connection degree.

### Gene body analysis and KEGG pathway enrichment analysis

Gene body analysis (GO) and KEGG pathway enrichment analysis were performed on the common targets of HTD and reflux esophagitis using David 6.8 database (https://david.ncifcrf.go/). The top 10 significantly enriched GO functions and the top 20 KEGG pathways were screened with *P* < 0.01. The online platform of microbioinformatics data analysis and visualization (http://www.Bioinformatics.com.cn) was used for data visualization processing to explain the biological functions and related signaling pathways of HTD in the treatment of NERD. Cytoscape 3.8.2 software was used to construct the network of “drug active components-potential target-pathway”, and the network topology analysis was carried out, and the core chemical components were analyzed by BC, CC and degree.

### Clinical Studies

#### Study design

This was a single-center, double-blinded, placebo-controlled randomized controlled trial (RCT) conducted at Tianjin Nankai Hospital. The study adhered to the principles outlined in the Declaration of Helsinki, Edinburgh 2000 edition. The final protocol (version 1.0, dated August 16, 2023) received approval from the Ethics Committee of Tianjin Nankai Hospital (approval number: NKYY-YW-IRB-2023-003-01). Furthermore, the trial is registered with the Chinese Clinical Trial Registry (registration number: ChiCTR2400081029).

We aimed to recruit 60 adult NERD patients with acid reflux or heartburn symptoms in the outpatient department of Gastroenterology of Tianjin Nankai Hospital from February 2024 to November 2024. After a screening period of 0–1 week, the eligible patients were randomly assigned to the TCM group (HTD) or the placebo group (TCM granule mimic), administered 5 g twice daily for 4 weeks and 2 weeks of follow-up. SAS 9.4 statistical software was used to generate the random number grouping table according to the number of cases and the random proportion. The experimental group and the control group were randomly coded in a ratio of 1: 1. The random number was used as the drug number.

HTD granules are produced by Tianjin Tasly Pharmaceutical Co. Ltd., including white and white, salvia miltiorrhiza, Helicoverpa, Fructus aurantii, Xiangfu, Tulip, and Pinellia. This TCM formula has obtained a Chinese invention patent (authorized notice No. CN 114377097B). In order to maintain the double-blinded condition, a TCM placebo with a similar appearance and taste was designed. The TCM placebo was prepared by Tianjin Tasly Pharmaceutical Co. Ltd. according to the preparation process of HTD. After adding the same type and an equal amount of excipient, its shape, color, gas, and taste were similar to those of HTD. Finally, 5% of the original unit of the treatment drug was added to correct the color and taste.

#### Patients

Our study objective was to enroll patients exhibiting characteristic symptoms of gastroesophageal reflux (such as acid reflux and heartburn).

Diagnostic criteria: The diagnostic criteria of Western medicine referred to the Chinese expert consensus on gastroesophageal reflux disease released in 2020. The TCM diagnostic criteria referred to the diagnosis of Qi stagnation and blood stasis syndrome in the Consensus on Integrated Traditional Chinese and Western Medicine Diagnosis and Treatment of Gastroesophageal Reflux Disease, issued by the Digestive Committee of Chinese Association of Integrated Traditional Chinese and Western Medicine in 2017.

A preliminary diagnosis of NERD can be made if the following two criteria are met: ① typical heartburn and reflux symptoms persist or recur for > 3 months and have a significant negative impact on the quality of life of patients: mild symptoms on > 2 days in a week, or moderate or severe symptoms on > 1 day in a week. ② No Barrett’s esophagus and esophageal mucosal damage were detected by gastroscopy. The gastroesophageal reflux disease questionnaire (GERD-Q) score ≥8 points can make a preliminary diagnosis.

#### Qi stagnation and blood stasis syndrome

Main symptoms: ① acid reflux for a long time; ② stabbing or pain behind the sternum, fixed position; ③ Dysphagia.

Secondary symptoms: ① belching; ② chest tightness; ③ hematemesis and hematochezia; ④ aggravation of the condition when the mood is poor.

Tongue pulse: the tongue color is dark or has ecchymosis, the tongue coating is white, and the pulse string is thin or astringent.

If the patient has two of the above main symptoms and two of the secondary symptoms, the diagnosis can be made by referring to the tongue pulse.

#### Inclusion criteria

The confirmed non-erosive reflux disease conformed to the diagnostic criteria of Western medicine:

1. Age of 18–65 years (including boundary value), regardless of the gender;
2. consistent with the diagnostic criteria of TCM qi stagnation and blood stasis syndrome;
3. The mean weekly reflux or heartburn Visual Analog Scale [VAS] score ≥3.0;
4. GERD-Q score ≥8;
5. Patients provided informed consent and were willing to receive the corresponding treatment.

#### Exclusion criteria

1. Reflux esophagitis was diagnosed by gastroscopy within the past year or combined with other serious esophageal complications, such as esophageal ulcer, hiatal hernia, Barrett’s esophagus, early esophageal cancer, or esophageal cancer;
2. previous surgery that possibly affected the esophageal or gastric function or gastric acid secretion; surgical procedures, such as abdominal surgery and vagotomy, which may have affected esophageal and gastric motility and gastric acid secretion, or were planned during the study;
3. previous rheumatic diseases that may have affected esophageal motility, such as scleroderma and Behcet’s disease, or present with a history of esophageal radiotherapy or esophageal cryotherapy;
4. patients with previous definite diagnosis of organic diseases of the digestive system, such as achalasia, Zolf-Ellison syndrome, inflammatory bowel disease, intestinal tuberculosis, intestinal ulcer, acute and chronic pancreatitis, liver cirrhosis, cholecystolithiasis with acute cholecystitis, other tumors of the digestive system, or peptic ulcer in the past 3 months;
5. The 13C breath test indicated current *H. pylori* infection before randomization;
6. patients with other definite systemic diseases affecting digestive tract function, such as hyperthyroidism or hypothyroidism, chronic renal insufficiency, autoimmune diseases, and diabetes.
7. patients with serious diseases of the heart, brain, liver, kidney, immune system, hematopoietic system, tumor, nervous system, or mental system (such as severe depression/anxiety/bipolar disorder);
8. concomitant drugs that affected gastrointestinal motility and function, such as antacids, antacids, prokinetic drugs, anticholinergic drugs, calcium channel blockers (except antihypertensive drugs), 5-HT3 receptor antagonists, antidepressants, and anxiolytic drugs, which could not be stopped during the study;
9. taking banned drugs during the run-in period;
10. pregnant or lactating women, patients who planned to have children within 1 month after enrollment;
11. allergic to the test drug and its ingredients;
12. suspected or indeed having a history of alcohol or drug abuse;
13. patients who participated in other clinical trials within 1 month before enrollment;
14. other patients considered by the investigator to be unsuitable for a clinical trial, such as a severe condition requiring emergency treatment or insufficient compliance to complete the study.

#### Study assessments

1. Efficacy
  ① VAS scores and response rates for clinical symptoms (such as reflux and heartburn)
    The VAS scores for primary clinical symptoms (such as acid regurgitation and heartburn) and the overall effectiveness rate were evaluated. Patients maintained a symptom diary throughout the study period. The participants rated the severity of their heartburn and acid reflux symptoms using a VAS (0–10 scale) at bedtime and documented these ratings in their symptom diaries. Evaluators collected daily scores at baseline, week 2, and week 4, and 6th week of follow-up and computed the average score for each symptom over the respective weeks.
    VAS score criteria:
    Score 0: no reflux or heartburn symptoms;
    Score 1–2: the symptoms were not obvious and found under the doctor’s reminder;
    Score 3–4: symptoms were obvious, but did not affect the lifestyle;
    Score 5–6: symptoms were more obvious but with little effect on the daily life;
    Score 7–8: symptoms were more obvious and had a greater impact on daily life;
    Score 9–10: symptoms are so pronounced that they seriously interfere with daily life, requiring supplementation with other medications.
    The response rate of any symptom was defined as a reduction of ≥50% in the average score of each symptom per week after treatment compared with the baseline value, and the response number of weeks was > 50% of the total number of weeks during the observation period.
    Overall clinical symptom relief rate: The total average score for clinical symptoms (such as reflux and heartburn) over the previous week is calculated, and the response is recorded as a decrease of 50% or more in the average total score post-treatment compared to baseline. If the number of weeks showing this response exceeds 50% of the total observation period, the treatment is considered to provide effective relief.
  ② GERD-Q scale scores
    GERD-Q is an internationally recognized and most widely applied special scale for GERD diagnosis. In addition to diagnosing GERD, it is possible to evaluate the impact of GERD on the quality of life and to monitor treatment effects with high precision.^[21]^ Trained assessors recorded assessments at the baseline, week 2, week 4, and 6th week of follow-up, filled out GERD-Q questionnaires, and calculated the total score of GERD-Q symptoms. The GERD-Q scale score after 4 weeks of treatment was considered the primary outcome.
  ③ TCM clinical syndrome and curative effect evaluation
    Based on the diagnostic criteria for Qi stagnation and blood stasis syndrome, primary and secondary symptoms, along with tongue and pulse characteristics, were graded using a standardized scale. Evaluations were conducted at baseline, week 2, week 4, and 6th week of follow-up. Following the “Guiding Principles for Clinical Research of New Chinese Medicine” ^[22]^ symptoms were classified into four grades: none, mild, moderate, and severe. Scores ranged from 0, 2, 4, and 6 points for primary symptoms and 0, 1, 2, and 3 points for secondary symptoms. Symptom scores were recorded at baseline, week 2, and week 4, and total score changes before and after treatment were assessed. The efficacy index was calculated as follows: efficacy index = (total score before treatment- total score after treatment) /total score before treatment × 100%.
    Clinical cure: reflux symptoms disappeared, efficacy index ≥ 95%.
    Remarkable effect: reflux symptoms mostly resolved with occasional occurrences that quickly subsided, 70%≤ efficacy index < 95%.
    Effective: reflux symptoms reduced but not resolved, 30% ≤ efficacy index < 70%.
    Ineffective: reflux symptoms persisted or worsened, efficacy index < 30%.
    Effective rate= (clinical cure + marked effect + effective) number/total number of cases in the group × 100%.
  ④ Quality-of-life score:
    Quality of life was evaluated using the Chinese version of the SF-36 health survey, covering nine dimensions: physical functioning, role limitations due to physical problems, bodily pain, general health, vitality, social functioning, role limitations due to emotional problems, and mental health. Assessments were conducted at baseline, week 4, and 6th week of follow-up.
  ⑤ HAD anxiety and depression scores
    The HAD was used to measure anxiety and depression levels at baseline, week 4, and 6th week of follow-up.
  ⑥ Recurrence indicators were followed up
    Patients with GERD-Q < 8 at week 4 were included in the recurrence rate calculation two weeks after treatment completion. The recurrence rate = the number of cases with GERD-Q≥8 at 2 weeks of follow-up after treatment/the number of cases with GERD-Q < 8 at 4 weeks of treatment ×100%.
2. Safety

Study drug safety and medication adherence were monitored through patient-reported adverse events, physical examination results, and laboratory tests at weeks 2 and 4 after randomization. An adverse drug reaction was defined as any unexpected adverse event occurring at any dose of the study drug, with a potential causal relationship that could not be ruled out. Medication compliance was defined as good if it was 80.0% or greater and 120.0% or less. The safety group included all patients who received at least one dose of the study drug and completed a follow-up safety assessment.

#### Sample size and statistical analysis

The sample size was calculated using a type I error rate of 0.05 and a statistical power of 80%. Data from a previous clinical study of the Huoxue Tongjiang recipe indicated a symptom improvement response rate of at least 65% with prior medical treatment,^[23]^ compared to an assumed 30% response rate in the placebo group. Based on these parameters, a minimum of 27 patients per group was required using a two-sided Fisher’s exact test at a significance level of 0.05. To account for a potential 15% dropout rate, the sample size was increased to 32 patients per group.

All statistical analyses were performed using SAS 9.4. Continuous variables were expressed as means ±standard deviations. After normality testing, inter-group comparisons were conducted using either the two-sample t-test or the Wilcoxon rank-sum test, while intra-group comparisons were performed using analysis of variance (ANOVA). Categorical data were analyzed using the χ^2^test or Fisher’s exact test, as appropriate. All statistical tests were two-sided, with *P* < 0.05 considered statistically significant.

## Results

### Activity screening and target collection of HTD

**Figure 1:**
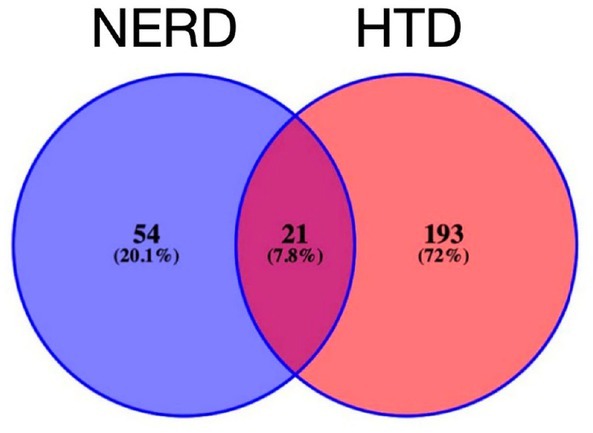
Network diagram of “compound component-target” of HTD. NERD, Non-erosive reflux disease; HTD, Huoxue Tongjiang Decoction.

Through TCMSP database, OB ≥ 30%, DL ≥ 0.18 were used to screen out components without corresponding targets. A total of 146 active components, such as spiranol, salvianal and β-sitosterol, were screened out, and 214 corresponding targets were obtained. Meanwhile, the obtained active ingredients and target information are imported into Cytoscape3.8.2 software to construct a “drug active ingredient-target” network, as shown in Figure 1.

### Screening of common targets of HTD and NERD

Retrieval of “NERD” in GeneCards database (https://www.Genecards.org/); Venny2.1 software was used to collect the intersection of the drug targets of HTD and the disease targets of NERD as the potential targets of HTD for the treatment of NERD. As a result, a total of 75 common targets were obtained.

### Protein interaction network construction of HTD for the treatment of NERD

**Figure 2:**
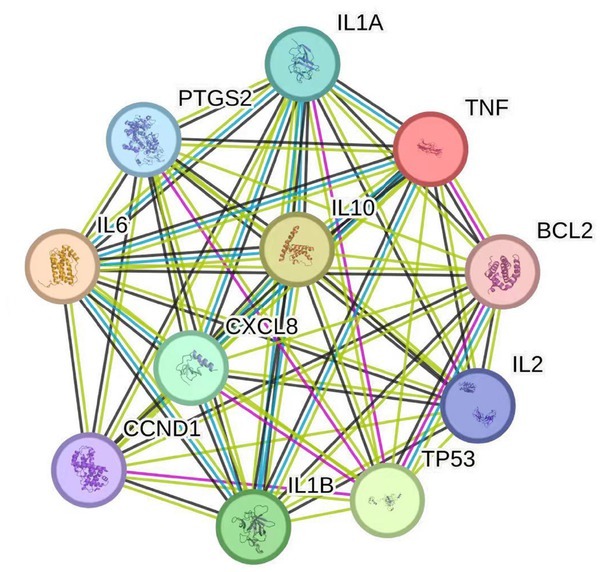
PPI network of potential targets of HTD. PPI, proton pump inhibitor; HTD, Huoxue Tongjiang Decoction.

The constructed PPI network has a total of 21 nodes and 169 edges, as shown in Figure 2. The larger the node is, the darker the color is, and the higher the degree is, indicating that these proteins occupy the network core. Topology analysis of PPI network was carried out, with BC greater than the median of 0.005014, CC greater than the median of 0.450867, and degree greater than the double median of 16.1, 17 core targets including STAT3, AKT1, and TNF were screened. These targets may play an important role in the treatment of NERD with HTD.

### Gene body analysis and KEGG pathway enrichment analysis

GO analysis and KEGG pathway enrichment analysis were performed on the common targets of HTD and NERD using David 6.8 database (https://david.ncifcrf.go/). The top 10 significantly enriched GO functions and the top 20 KEGG pathways were screened with *P* < 0.01. The online platform of micro-bioinformatics data analysis and visualization (http://www.Bioinformatics.com.cn) was used for data visualization processing to explain the biological functions and related signaling pathways of HTD in the treatment of NERD.

**Figure 3:**
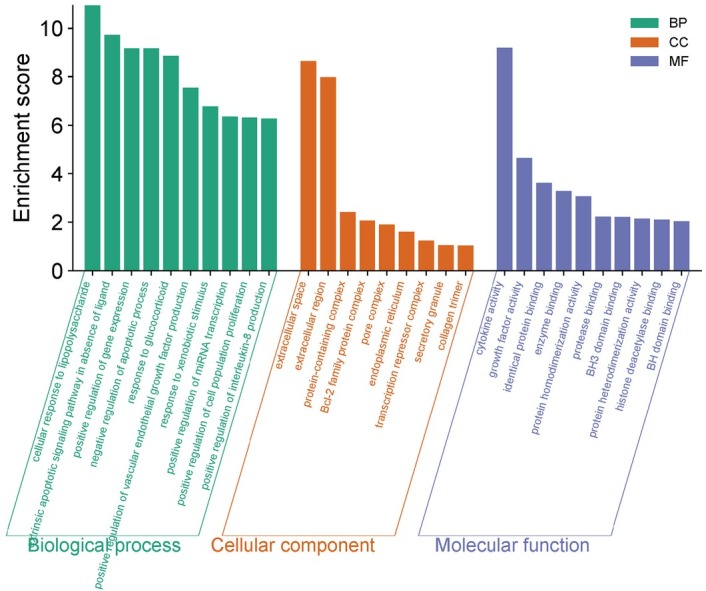
GO function analysis of potential targets of HTD for NERD. GO, Gene body; HTD, Huoxue Tongjiang Decoction; NERD, Non-erosive reflux disease.

GO analysis showed that 146 potential targets of HTD could regulate 571 biological processes. BP, mainly including positive regulation of transcription from RNA polymerase II promoter and negative regulation of apoptosis process Apoptotic process, response to drugs, and positive regulation of cell proliferation Apoptotic process, signal transduction, *etc*.; The cell consists of 53 cellular component, involving nucleus, cytoplasm, nucleosome, mitochondrion, *etc*. 87 Molecular functions (MF), It includes protein binding, enzyme binding, transcription factor binding and protein kinase binding, cytokine activity, *etc*. According to *P* < 0.01, the top 10 items were selected for visual analysis, as shown in Figure 3.

**Figure 4:**
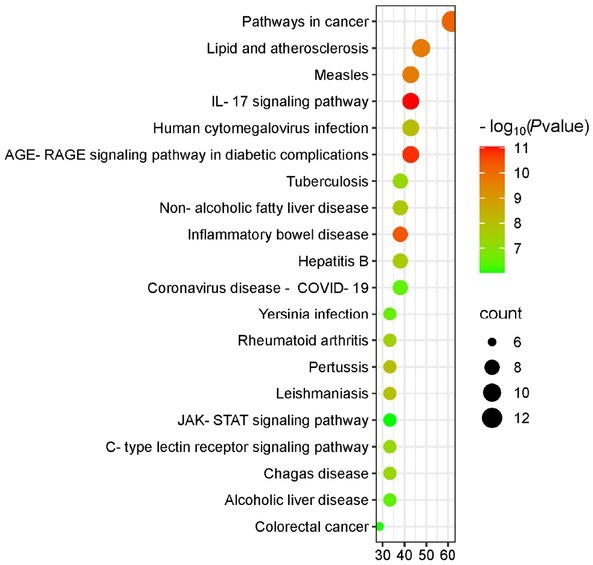
KEGG pathway analysis of potential targets of HTD for NERD. HTD, Huoxue Tongjiang Decoction; NERD, Non-erosive reflux disease.

KEGG pathway enrichment analysis results showed that potential targets of HTD were involved in the regulation of 106 pathways. According to *P* < 0.01, the top 20 pathways were selected for visualization, as shown in Figure 4. These Pathways include IL-17 signaling Pathway, C-type lectin receptor signaling Pathway, and JAK-STAT signaling pathway Signaling Pathway, *etc*.

### “Active component-target-pathway” network construction and topology analysis

**Figure 5:**
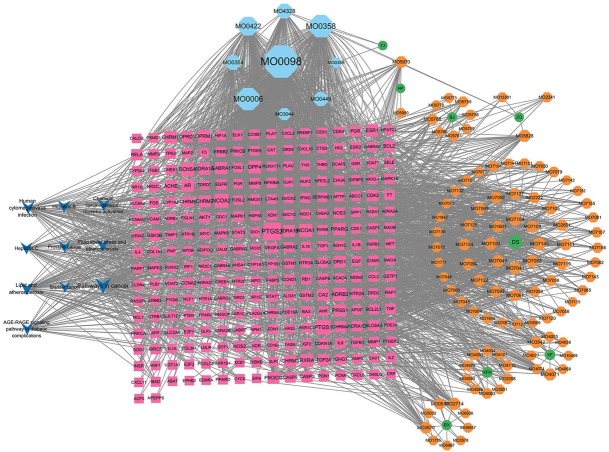
HTD “drug active component-potential target-pathway” network diagram. HTD, Huoxue Tongjiang Decoction.

Cytoscape 3.8.2 software was used to construct the network of “drug active components-potential target-pathway”, and the network topology analysis was carried out, and the core chemical components were analyzed by BC, CC and degree. The result is a network with 212 nodes and 835 edges, as shown in Figure 5. Network Analyzer was used to analyze the Network, and BC was greater than the median 0.0079, CC was greater than the median 0.385036, and degree was greater than the median 2 times 7. The 20 core chemical components in the Network were screened, as shown in Table 1. In addition, 67 potential targets were linked to quercetin, 36 to luteolin, 22 to kaempferol, 18 to baicalein, and 14 to naringin. These active components with high degree values may be the core components in the treatment of NERD.

**Table 1:** Topological properties of core components in the “active ingredient- target- pathway” network

TCMSP-ID	Compound	Source	Connection degree	Interface	Tightness
MOL000098	quercetin	Cyperi spiralis rhizoma, Cyperi	284	0.3204850536109155	0.503968253968254
MOL000358	beta-sitosterol	Pinellia ternata, Cyperi rhizoma, Curcuma aurantii, Aurantii fructus	160	0.03332135029735821	0.39278350515463917
MOL000006	luteolin	Salvia miltiorrhiza, Cyperi and Rhododendron rhizoma, amurense	159	0.0672134956818226	0.41413043478260875
MOL000422	kaempferol	Cyperi spiralis rhizoma, Cyperi	115	0.06074930166914527	0.41593886462882096
MOL004328	naringenin	Curcuma fructus and Aurantii	67	0.08729228942312925	0.3871951219512195
MOL000354	isorhamnetin	Cyperi spiralis rhizoma, Cyperi	63	0.0229986032904221	0.39036885245901637
MOL000449	Stigmasterol	Pinellia, Cyperi rhizoma	59	0.03833331456260495	0.3871951219512195
MOL007154	tanshinone IIa	Salvia miltiorrhiza	38	0.038851725348196836	0.39278350515463917
MOL007145	salviolone	Salvia miltiorrhiza	36	0.04353665217980667	0.38640973630831643
MOL002714	baicalein	Pinellia	33	0.04719517748627417	0.39036885245901637
MOL003044	Chryseriol	Cyperi spiralis rhizoma, Cyperi	33	0.0031503991416940006	0.37797619047619047
MOL007049	4-methylenemiltirone	Salvia miltiorrhiza	32	0.010877454082099862	0.39117043121149897
MOL007100	dihydrotanshinlactone	Salvia miltiorrhiza	32	0.008815978771460405	0.38640973630831643
MOL004071	Hyndarin	Salvia miltiorrhiza	30	0.015800655620362064	0.381
MOL007088	cryptotanshinone	Salvia miltiorrhiza	30	0.013698188469045712	0.39197530864197533
MOL007041	2-isopropyl-8-methylphenanthrene-3, 4-dione	Salvia miltiorrhiza	30	0.006677397641838021	0.3879837067209776
MOL005828	nobiletin	Zhike	29	0.03103418081057599	0.3871951219512195
MOL007108	isocryptotanshi-none	Salvia miltiorrhiza	29	0.006072557452681538	0.3879837067209776
MOL007124	neocryptotanshinone II	Salvia miltiorrhiza	27	0.005550577895491441	0.3840725806451613
MOL007098	deoxyneocryptotanshinone	Salvia miltiorrhiza	27	0.005550577895491441	0.3840725806451613

### Allocation of patients

**Figure 6:**
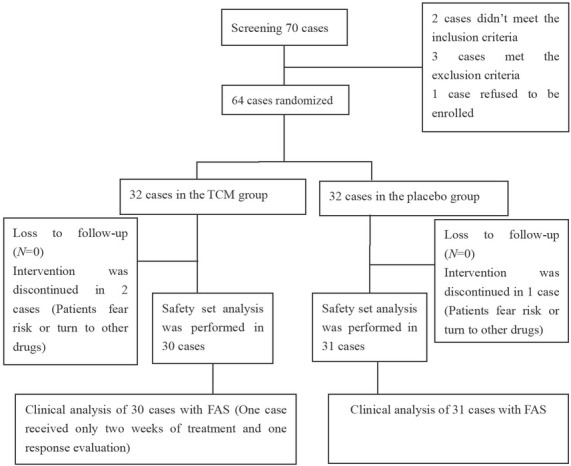
Depiction of the flow diagram of clinical trial. TCM, traditional Chinese medicine; FAS, full analysis set.

Seventy patients were screened, with six excluded during the screening process. The remaining 64 patients were randomly assigned to either the treatment group (32 patients) or the placebo group (32 patients) (Figure 6). Three patients were excluded from the safety analysis due to concerns about potential risks and switching to alternative medications (two from the treatment group and one from the placebo group); none of these patients received any study medication. A total of 61 patients who received at least one dose of the study drug and completed at least one post-baseline efficacy assessment were included in the full analysis set (FAS), comprising 30 patients in the treatment group and 31 in the placebo group. One patient discontinued the study after two weeks of treatment, completing only one efficacy assessment. The PPS calculation results were consistent with the FAS results; only the FAS results are provided in this result.

### Demographics and clinical characteristics

The demographic and baseline characteristics of the subjects, including sex, age, ethnicity, height, weight, marital status, and history of smoking and alcohol consumption, were well-balanced between the treatment and placebo groups (Table 2).

**Table 2:** Demographic characteristics of subjects with nonerosive esophageal reflux disease

Statistical item	TCM group (*N* = 30)	Placebo group (*N* = 31)	Sum up (*N* = 61)	*P*
Gender, *n* (%)				0.0143
Male	17 (56.7)	8 (25.8)	25 (41.0)	
Female	13 (43.3)	23 (74.2)	36 (59.0)	
Age (years, ± s)	47.2 ± 10.1	46.0 ± 10.9	46.6 ± 10.5	0.6753
Ethnic groups, *n* (%)				0.9812
Han nationality	29 (96.7)	30 (96.8)	59 (96.7)	
Other	1 (3.3)	1 (3.2)	2 (3.3)	
Height (cm, ± s)	168.9 ± 7.4	166.5 ± 7.1	167.7 ± 7.3	0.2110
Weight (kg, ± s)	65.7 ± 9.2	65.1 ± 11.0	65.4 ± 10.1	0.8259
Marital status, *n* (%)				0.9450
Married	27 (90.0)	29 (93.5)	56 (91.8)	
Unmarried	2 (6.7)	2 (6.5)	4 (6.6)	
Other	0	0	0	
Reproductive status, *n* (%)				0.3796
Nanparous	2 (6.7)	4 (12.9)	6 (9.8)	
Childbearing	20 (66.7)	18 (58.1)	38 (62.3)	
Smoking history, *n* (%)				0.8388
Never smoking	27 (90.0)	28 (90.3)	55 (90.2)	
Occasional smoking	2 (6.7)	1 (3.2)	3 (4.9)	
Regular smoking	1 (3.3)	1 (3.2)	2 (3.3)	
Quit smoking	0	0	0	
Drinking history, *n* (%)				0.2464
Never drinking	28 (93.3)	26 (83.9)	54 (88.5)	
Occasional drinking	2 (6.7)	5 (16.1)	7 (11.5)	
Regular drinking	0	0	0	
Quit drinking	0	0	0	
Other	0	0	0	

TCM, traditional Chinese medicine.

### Effective rate of total symptoms and major symptoms

In this study of NERD patients, the efficacy of TCM was compared to a placebo. Clinical response rates to TCM were assessed through the remission of clinical symptoms and the reduction of specific symptoms such as heartburn and acid reflux at different intervals: the 2nd, 4th, and 6th weeks of treatment and follow-up. Results indicated that the overall remission rates of clinical symptoms in the TCM group were 43.3%, 73.3%, and 73.3% at these respective times, which were significantly higher compared to the placebo group’s consistent 16.1% at all three points (*P* = 0.0199, *P* < 0.0001, *P* < 0.0001 respectively, Table 3). In terms of symptom-specific relief, the TCM group experienced notable improvements. By the 4th and 6th weeks, 66.7% of TCM recipients saw alleviation of reflux symptoms, a significant contrast to the 12.9% in the placebo group (*P* < 0.0001). No statistically significant difference was observed at the 2nd week (*P* = 0.1031). Relief rates for heartburn in the TCM group were 43.3%, 63.3%, and 76.7% at the 2nd, 4th, and 6th weeks, respectively, demonstrably superior to the placebo’s consistent 16.1% at these same intervals (*P* = 0.0199, *P* = 0.0002, *P* < 0.0001 respectively, Table 4).

**Table 3:** Comparison of effective rate of total symptoms between the two groups [*n* (%)]

	TCM group	Placebo group	*P*
Week 2	13 (43.3)	5 (16.1)	0.0199
Week 4	22 (73.3)	5 (16.1)	<0.0001
Week 6	22 (73.3)	5 (16.1)	<0.0001

TCM, traditional Chinese medicine.

**Table 4:** Comparison of effective rate of major symptom between the two groups [*n* (%)]

	TCM group	Placebo group	*P*
Reflux symptom response rate			
Week 2	9 (30.0)	4 (12.9)	0.1031
Week 4	20 (66.7)	4 (12.9)	<0.0001
Week 6	20 (66.7)	4 (12.9)	<0.0001
Heartburn symptoms response rate			
Week 2	13 (43.3)	5 (16.1)	0.0199
Week 4	19 (63.3)	5 (16.1)	0.0002
Week 6	23 (76.7)	5 (16.1)	<0.0001

TCM, traditional Chinese medicine.

### VAS score of major symptom comparisons

Additionally, we analyzed the weekly average VAS scores for both reflux and heartburn symptoms. During follow-up at weeks 2, 4, and 6, the TCM group demonstrated significant symptom score reductions in heartburn (from 3.33 ± 1.724 to 2.03 ± 1.607) and reflux (from 3.75 ± 1.599 to 2.25 ± 1.546), all reaching statistical significance (*P* < 0.05). Compared to 4.17 ± 2.215 and 4.22 ± 2.257 at weeks 4 and 6, the TCM group’s reflux scores were significantly lower (*P* < 0.05). Similarly, the heartburn scores significantly decreased compared to the placebo at 4.32 ± 2.015, 3.98 ± 2.059, and 4.25 ± 2.014 at the respective weeks. However, there was no statistically significant difference in the reflux scores between the groups at week 2. In conclusion, the TCM group displayed substantially greater improvement across all main symptoms of NERD compared to the placebo group with statistical significance, as detailed in Table 5.

**Table 5:** Comparison of score of major symptom between the two groups [± s (CI 95%)]

Symptom	Time	TCM group(*N* = 30)	Placebo group(*N* = 31)	95%CI	*P*
	Week0	5.1 ± 1.7 (4.4, 5.8)	5.0 ± 2.2 (4.3, 5.7)	(-0.8, 1.2)	0.7382
Reflux	Week2	3.8 ± 1.6 (3.1, 4.5)	4.5 ± 2.2 (3.8, 5.2)	(-1.7, 0.3)	0.1458
	Week4	2.0 ± 1.3 (1.3, 2.7)	4.2 ± 2.2 (3.5, 4.8)	(-3.1,-1.3)	<0.0001
	Week6	2.3 ± 1.6 (1.5, 3.0)	4.2 ± 2.3 (3.5, 4.9)	(-3.0,-1.0)	0.0002
	Week0	4.9 ± 1.3 (4.3, 5.5)	5.0 ± 1.8 (4.4, 5.5)	(-0.9, 0.7)	0.8735
Heartburn	Week2	3.3 ± 1.7 (2.6, 4.0)	4.3 ± 2.0 (3.7, 5.0)	(-2.0,-0.1)	0.0434
	Week4	1.8 ± 1.5 (1.1, 2.5)	4.0 ± 2.1 (3.3, 4.6)	(-3.1,-1.2)	<0.0001
	Week6	2.0 ± 1.6 (1.4, 2.7)	4.3 ± 2.0 (3.6, 4.9)	(-3.2,-1.3)	<0.0001

TCM, traditional Chinese medicine.

### GERD-Q scale score comparisons

The GERD-Q scores for the TCM group demonstrated a significant reduction at the 2nd (7.9 ± 2.29, *P* = 0.0015), 4th (6.2 ± 2.07, *P* < 0.0001), and 6th weeks (6.8 ± 2.40, *P* < 0.0001) compared to baseline. In contrast, the placebo group’s scores at weeks 2 (9.4 ± 2.83, *P* = 0.0280), 4 (8.9 ± 2.55, *P* < 0.0001), and 6 (8.9 ± 2.59, *P*= 0.0016) did not show a statistically significant difference from baseline (*P* > 0.05), underscoring a superior improvement in the GERD-Q scale scores in the TCM group relative to the placebo group. These findings are detailed in Table 6, which presents a comparison of the total GERD-Q scores between the two groups.

**Table 6:** Comparison of total scores of symptom (GERD-Q Scores) between the two groups [± s (CI 95%)]

Time	TCM group	Placebo group	95%CI	*P*
	(*N* = 30)	(*N* = 31)		
Week0	9.6±1.4 (9.0, 10.2)	9.7±1.8 (9.1, 10.3)	(-0.9, 0.7)	0.7929
Week2	7.9±2.3 (7.0, 8.9)	9.4±2.8 (8.5, 10.4)	(-2.8,-0.2)	0.0280
Week4	6.2±2.1 (5.3, 7.0)	8.9±2.6 (8.0, 9.7)	(-3.9,-1.5)	<0.0001
Week6	6.8±2.4 (5.9, 7.7)	8.9±2.6 (8.0, 9.8)	(-3.4,-0.9)	0.0016

TCM, traditional Chinese medicine.

### Remission rate of total TCM syndrome score

The effectiveness rates of the TCM group at the 2nd, 4th, and 6th weeks of treatment and during follow-up were 56.7%, 86.7%, and 76.7%, respectively. In contrast, the placebo group exhibited effectiveness rates of 16.1%, 25.8%, and 41.9%, respectively. Chi-square test analyses demonstrated that the TCM group consistently outperformed the placebo group in terms of effectiveness throughout each observational period (all *P* < 0.05, Table 7).

**Table 7:** Comparison of effective rate between the two groups [*n* (%)]

Time	Group	Recover	Remarkable effect	Effective	Ineffective	Total effective rate	*P*
	TCM Group						
	(*N* = 30)	1 (3.3)	1 (3.3)	15 (50.0)	13 (43.3)	56.7%	
Week2	Placebo group						0.0085
	(*N* = 31)	1 (3.2)	0 (0.0)	4 (12.9)	26 (83.9)	16.1%	
	TCM Group						
Week4	(*N* = 30)	8 (26.7)	5 (16.7)	13 (43.3)	4 (13.3)	86.7%	<0.0001
	Placebo group						
	(*N* = 31)	2 (6.5)	2 (6.5)	4 (12.9)	23 (74.2)	25.8%	
	TCM group						
Week6	(*N* = 30)	4 (13.3)	7 (23.3)	12 (40.0)	7 (23.3)	76.7%	0.0293
	Placebo group						
	(*N*=31)	3 (9.7)	3 (9.7)	7 (22.6)	18 (58.1)	41.9%	

TCM, traditional Chinese medicine.

### Quality-of-life comparisons

After four weeks of treatment, the physical pain scores of patients in the TCM group were significantly higher than those in the placebo group (81.0 ± 11.57 *vs*. 74.4 ± 10.65), with a statistically significant difference (*P* = 0.0234). No statistical differences were observed in other indicators between the two groups (all *P* > 0.05). At six weeks, the TCM group exhibited higher scores across various indicators including physical pain, energy, social function, and mental health, compared to the placebo group, with all differences being statistically significant (*P* < 0.05). Overall, the improvement in these indicators was more pronounced in the TCM group than in the placebo group, as detailed in Table 8.

**Table 8:** Comparison of QOL between the two groups (± s)

	Time	TCM group (*N* = 30)	Placebo group (*N* = 31)	*P*
Physiological function	Week0	89.0 ± 15.3	91.6 ± 10.7	0.4418
	Week4	92.1 ± 11.5	93.4 ± 9.7	0.6315
	Week6	93.3 ± 10.5	93.1 ± 10.6	0.9384
Physiological function	Week0	71.7 ± 40.9	79.0 ± 38.2	0.4700
	Week4	83.6 ± 32.2	75.8 ± 39.0	0.4028
	Week6	82.8 ± 33.5	76.6 ± 37.6	0.5075
Body pain	Week0	74.3 ± 12.4	71.3 ± 14.	0.3830
	Week4	81.0 ± 11.6	74.4 ± 10.7	0.0234
	Week6	83.1 ± 9.3	77.0 ± 11.7	0.0302
General health status	Week0	59.4 ± 17.5	61.5 ± 20.9	0.6698
	Week4	66.9 ± 19.9	60.6 ± 19.5	0.2238
	Week6	70.3 ± 18.6	62.9 ± 19.7	0.1401
Energy	Week0	66.0 ± 17.2	64.2 ± 19.8	0.7053
	Week4	71.7 ± 17.9	64.2 ± 20.6	0.1374
	Week6	77.4 ± 15.5	67.7 ± 18.1	0.0305
Social function	Week0	70.0 ± 19.6	69.76 ± 19.8	0.9619
	Week4	79.7 ± 17.8	70.6 ± 18.4	0.0548
	Week6	83.6 ± 14.2	72.18 ± 19.0	0.0110
Emotional function	Week0	53.3 ± 50.0	68.8 ± 39.4	0.1832
	Week4	73.6 ± 45.8	72.0 ± 43.1	0.8951
	Week6	87.4 ± 30.1	72.0 ± 43.1	0.1185
Mental health	Week0	64.5 ± 15.8	59.5 ± 18.5	0.2563
	Week4	71.7 ± 19.1	63.7 ± 17.2	0.0937
	Week6	76.8 ± 15.8	67.0 ± 18.7	0.0318
Changes in health	Week0	51.7 ± 20.7	54.0 ± 21.5	0.6633
	Week4	62.1 ± 22.9	54.0 ± 18.4	0.1367
	Week6	67.2 ± 26.8	55.6 ± 20.1	0.0620

TCM, traditional Chinese medicine.

### HDA scale score

By the fourth week of treatment and the sixth week of follow-up, patients in both groups exhibited a reduction in anxiety and depression scores, although the differences were not statistically significant (*P* > 0.05 for both). Subsequent analysis revealed that the decrease in depression scores from baseline to week four was significantly greater in the TCM group compared to the placebo group (*P* = 0.0490). Therefore, the improvement in depression scores may be more pronounced in the TCM group than in the placebo group, as indicated in Table 9.

**Table 9:** Comparison of HAD score between the two groups (± s)

	Time	TCM group (*N* = 30)	Placebo group (*N* = 31)	*P*
Anxiety	Week0	6.5 ± 4.4	6.6 ± 4.8	0.9027
	Week4	5.3 ± 3.9	6.8 ± 4.9	0.1976
	Week6	4.2 ± 3.9	6.1 ± 4.8	0.1022
Depression	Week0	5.9 ± 5.0	6.4 ± 4.8	0.7001
	Week4	4.0 ± 4.4	6.1 ± 4.6	0.0768
	Week6	3.4 ± 4.0	5.5 ± 4.6	0.0647

TCM, traditional Chinese medicine.

### Rate of recurrence

The recurrence rate was 27.3% (6/22) in the TCM group, significantly lower than 40.0% (4/10) in the placebo group (*P* < 0.05).

### Adverse reactions

As for the safety and tolerability of the study product and placebo, no adverse drug reactions were reported throughout the study period. Blood routine, urine routine, liver and kidney function, electrolyte and thyroid function after treatment were not significantly abnormal compared with the baseline before treatment. One case in the Chinese medicine group showed positive occult blood in stool routine after treatment, but this case did not show any new symptoms, and there were no abnormal changes in routine blood routine, heart rate, blood pressure, *etc*., which was recorded as Adverse events. In addition, one patient in the placebo group had uncontrolled heartburn symptoms due to acid reflux and was temporarily treated with aluminum-magnesium carbonate tablets according to the study design. There was no significant difference in the incidence of adverse events between the two groups, as indicated in Table 10.

**Table 10:** Adverse events during the study

Events	TCM group (*N* = 30)	Placebo group (*N* = 31)	*P*
Total	1 (3.3%)	1 (3.23%)	0.9813
Blood routine examination	0	0	-
Liver function	0	0	-
Renal function	0	0	-
Thyroid function	0	0	-
Urinalysis	0	0	-
Stool routine	1 (3.3%)	0	0.3094
Remedial drug	0	1 (3.23%)	0.2418

TCM, traditional Chinese medicine.

## Discussion

GERD is a prevalent chronic digestive disorder characterized by reflux and heartburn. Atypical symptoms, including chest pain, upper abdominal burning, pain, distension, belching, and extra-esophageal manifestations, are also common. NERD is the most frequent clinical subtype of GERD, with a multifactorial etiology involving esophageal and gastrointestinal motility disorders, acid and weak acid reflux, bile reflux, mixed gas-liquid reflux, visceral hypersensitivity, psychological factors, gut microbiota dysbiosis, and disturbances in brain-gut interactions.^[9, 12, 13, 14]^ Current GERD management primarily relies on acid-suppressive therapies, particularly PPIs. However, studies indicate that symptom relief with PPI treatment is achieved in only 56%–76% of patients with GERD, with even lower response rates observed in NERD cases.^[7, 24, 25]^ An epidemiological study of 21, 010 patients with typical reflux symptoms revealed that approximately 50% were diagnosed with NERD.^[26]^ Notably, up to 70% of patients with GERD show no esophageal mucosal damage (NERD) during endoscopy, underscoring the clinical significance of effective NERD management. A large survey conducted by the American Gastroenterology Association reported that over 55% of patients with GERD continue to experience significant impairments in quality-of-life despite PPI therapy.^[27]^ These findings highlight the urgent need to develop novel, effective therapeutic approaches for NERD management.

TCM emphasizes the integration of disease and syndrome, addressing both symptoms and underlying conditions while treating the mind and body holistically. Guided by a holistic approach and syndrome differentiation, TCM demonstrates significant advantages in alleviating symptoms and improving patients’ quality of life. TCM treatments often involve multiple therapeutic targets and mechanisms of action, enabling them to address the complex pathophysiology of NERD and effectively relieve clinical symptoms. Through clinical practice, TCM aims to refine the appropriate patient populations, dosage, efficacy profiles, and clinical benefits, ultimately standardizing treatment prescriptions. The Huoxue Tongjiang recipe was developed based on TCM theory and modern gastrointestinal motility research. It contains ingredients such as *Angelica sinensis*, *Salvia miltiorrhiza*, *Fructus aurantii*, *Cyperus rotundus* (Xiangfu), *Tulipa edulis* (Tulip), *Pinellia ternata*, and others. This formulation is known to activate blood circulation, promote tissue repair, reduce gastric acid secretion, and alleviate pain. Previous experimental studies demonstrated that HTD reduces gastric acid secretion, increases the number and activity of interstitial cells of Cajal *via* the upregulation of c-kit/SCF expression, enhances lower esophageal sphincter pressure, and improves gastric emptying. Additionally, it strengthens the intestinal mucosal barrier by modulating the intestinal flora, reduces inflammatory cytokines such as IL-6, TNF-α, and IL-1β, alleviates esophageal mucosal immune inflammation, and improves esophageal visceral hypersensitivity.^[16, 17, 18, 19, 20]^ This study aims to further validate the clinical efficacy of HTD in the treatment of patients with NERD. Network pharmacological analysis revealed the multi-dimensional mechanism of HTD, in the treatment of NERD. Firstly, the core components such as quercetin and baicalin in HTD, can alleviate the inflammatory response of the esophageal mucosa by inhibiting the TNF signaling pathway (such as down-regulating the expression of TNF-α and IL-6), which is consistent with the results in animal experiments that HTD, reduces the levels of IL-1β and TNF-α in esophageal tissues.^[28]^ Secondly, the regulation of the HIF-1 signaling pathway may enhance the mucosal hypoxia adaptation ability, promote angiogenesis and tissue repair,^[29]^ thereby improving the mucosal barrier function of patients with NERD. Furthermore, the participation of the JAK-STAT pathway suggests that HTD, may alleviate visceral hypersensitivity by regulating immune cell differentiation (such as Th17/Treg balance).^[30]^ It is notable that the effect of HTD, on the TP53 target may involve the regulation of apoptosis and the inhibition of excessive apoptosis of esophageal epithelial cells, while the upregulation of IL-10 may maintain mucosal homeostasis through the anti-inflammatory pathway. These multi-pathway synergies not only explain the clinical effect of HTD, in rapidly relieving symptoms (such as a significant reduction in GERD-Q score after 4 weeks), but also provide a molecular basis for its long-term reduction in the recurrence rate.

In this study, 61 patients were administered HTD, resulting in a 35.4% reduction in total symptom scores and an overall symptom efficacy rate of 86.7%. The response rates for reflux and heartburn symptoms were 76.7% and 73.3%, respectively. In contrast, the placebo group showed only an 8.2% reduction in total symptom scores, a symptom efficacy rate of 25.8%, and main symptom response rates of 16.1%. No adverse events were reported, highlighting the significant efficacy and safety of TCM treatment compared to placebo. Furthermore, patients in the TCM group experienced notable improvements in quality of life, particularly in terms of body pain, compared to the placebo group. This study observed that NERD patients who were administered TCM exhibited varied but significant reductions in their HAD depression scores compared to those in the placebo group. The comparison before and after treatment underscored a statistically significant difference, suggesting that HTD is effective in ameliorating depressive symptoms in patients. These findings suggest that HTD may alleviate depressive symptoms in patients with NERD. Previous studies have demonstrated that depression is closely associated with reflux symptoms, increasing the risk of reflux by 1.7 times (95% CI: 1.4–2.1).^[31]^ Moreover, depression has been identified as a factor influencing treatment outcomes, with evidence indicating its role in PPI treatment failure (*P* = 0.005).^[32]^ Finally, the study found that the recurrence rate of symptoms two weeks post-treatment was significantly lower in the TCM group compared to the placebo group. The results presented above are consistent with the pharmacological mechanism observed in prior animal studies treating NERD. The classic Western medical treatment for NERD mainly involves acid-suppressing drugs (proton pump inhibitors PPIs or H^2^ receptor antagonists), and can be supplemented with gastrointestinal motility drugs. Although PPIs can effectively inhibit gastric acid secretion, 30%-40% of patients still do not respond to PPIs, especially those with non-acid reflux and weak acid reflux.^[33]^ In addition, long-term use of PPIs may cause side effects such as osteoporosis, intestinal flora imbalance and nutrient absorption disorders. Recent studies have pointed out that the pathological mechanism of NERD is complex, involving multiple factors such as impaired esophageal mucosal barrier function, persistent immune inflammatory response, and abnormal brain-gut interaction.^[34]^ Single-target drug treatment is difficult to completely control the symptoms. In contrast, after HTD treatment, not only is the remission rate of reflux and heartburn symptoms in NERD patients as high as 73.3%, It also has the comprehensive effects of regulating gastroesophageal motility, improving mucosal blood flow, and reducing visceral hypersensitivity. Moreover, the recurrence rate after drug withdrawal is lower than that of traditional Western medicine (27.3% *vs*. 40.0%).

This study has several limitations. First, it is a single-center study with a small sample size. Future research should involve multi-center, increase the sample size, and extend the follow-up period to assess the efficacy of HTD across diverse populations with varying regional and environmental factors. Second, the latest Rome IV criteria categorize the NERD phenotype into true NERD with pathological reflux, reflux hypersensitivity, and functional heartburn without pathological reflux. Due to practical constraints and patient acceptance, 24-hour pH-impedance monitoring was not performed in this study. As a result, patients with non-pathological acid reflux could not be excluded. While the proportion of such patients is likely low, individuals with functional heartburn and reflux hypersensitivity, who also present with heartburn and acid reflux symptoms, may have been included. The National Medical Products Administration of China recommends that non erosive reflux disease often overlaps with functional heartburn and high sensitivity to reflux in clinical diagnosis and treatment, all of which show no signs of esophageal mucosal damage under endoscopy but may present symptoms of heartburn and reflux. The purpose of its treatment is all symptom improvement. According to the traditional Chinese medicine method of “treating different diseases with the same treatment”, starting from the actual clinical diagnosis and treatment, for traditional Chinese medicine treatments that improve symptoms similar to endoscopic negative gastroesophageal reflux disease, it is not required to exclude functional heartburn and high sensitivity to reflux. Additionally, this study found that there was no significant improvement in anxiety scores, contradicting the initial expectations. This may be attributed to the brevity of the treatment course. Future studies could consider extending the duration of treatment and the follow-up period post-withdrawal. Such adjustments would allow for more detailed observation of the drug’s efficacy over different timeframes, thereby enhancing clinical applicability.

This study specifically targeted GERD-like symptoms in patients with negative endoscopy findings, employing a double-blind, placebo-controlled superiority design. This rigorous methodology enhances the reliability and validity of the results. By integrating traditional Chinese medicine theory, clinical experience, and evidence-based research, the study establishes a solid theoretical and scientific foundation for developing new TCM treatments for non-erosive reflux disease.

Conclusion: HTD has been shown to effectively alleviate clinical symptoms, including reflux, heartburn, and retrosternal pain, among patients with NERD. Additionally, it enhances patients’ quality of life and decreases recurrence rates. In addition, there are no security issues.
